# Changes in Eating Behavior Among Children with Overweight or Obesity: Results of a Nutritional Intervention

**DOI:** 10.3390/nu18061012

**Published:** 2026-03-23

**Authors:** Luana de Paula Ivnuk, Ádelin Olivia Lopes Joly Rodrigues, Isabela Cristina Santos Freire de Paula, Carlos Henrique Pereira, Marina Amaro da Rocha Matuguma, Gustavo Hermes Soares, Renata Iani Werneck, Juliana Schaia Rocha Orsi

**Affiliations:** 1 Postgraduate Program in Dentistry, Pontifícia Universidade Católica do Paraná, Curitiba 80215-901, PR, Brazil; adelin.joly@pucpr.edu.br (Á.O.L.J.R.); isabela.freire@pucpr.edu.br (I.C.S.F.d.P.); carlos.pereira3@pucpr.edu.br (C.H.P.); marina.amaro@pucpr.edu.br (M.A.d.R.M.); renata.iani@pucpr.br (R.I.W.); juliana.orsi@pucpr.br (J.S.R.O.); 2 Australian Research Centre for Population Oral Health, Adelaide Dental School, University of Adelaide, Adelaide, SA 5005, Australia; gustavo.hermessoares@adelaide.edu.au

**Keywords:** nutrition, health education, eating behavior

## Abstract

Background/Objectives: This study aimed to explore perceived changes in the eating behavior of children with overweight and obesity following a nutritional intervention, considering the perspectives of children and their families participating in a specialized health promotion program. Methods: This qualitative study included five children aged 7 to 12 years and four family members enrolled in the *ProSaúde Kids* Program in southern Brazil. The study comprised three stages: initial semi-structured interviews; nine interactive nutritional workshops conducted from July to November 2024 with active family participation; and final interviews after the intervention. Recordings were transcribed and analyzed in ATLAS.TI^®^ using the collective subject discourse approach, guided by Bronfenbrenner’s Ecological Systems Theory. Results: The intervention was associated with reported changes in perceptions and eating practices. Families described greater involvement of children in food decisions, perceived reductions in consumption of ultra-processed foods, and reported reorganization of eating routines. Children expressed increased critical awareness of food-related media content and greater appreciation of body diversity. Improvements in the quality of homemade school snacks suggested meaningful family engagement, even in the absence of direct school involvement. Conclusions: The workshops were described as encouraging reflection and supporting changes in attitudes and practices. Overall, the findings indicate potential positive shifts in eating behavior, greater awareness of food choices, and enhanced family participation in the behavior-change process.

## 1. Introduction

Childhood obesity is recognized as one of the major health burdens in Brazil, affecting 13% of children aged 0–5 years and 31.65% of those aged 6–10 years, according to BMI-for-age criteria [1]. This situation is particularly concerning due to its strong association with the early onset of metabolic syndrome—a cluster of interrelated metabolic disorders that include insulin resistance, dyslipidemia, hypertension, and central obesity—all of which significantly increase the risk of cardiovascular diseases, type 2 diabetes, and other chronic complications throughout life [2]. Among the factors considered globally essential for the prevention and control of this condition are physical activity and dietary habits [3,4].

Eating behavior is defined as a set of individual reactions to the environment and circumstances in which individuals are embedded [5]. Numerous factors influence eating behavior and may contribute to the increasing rates of overweight and obesity. These include external factors—such as social and family units, social and cultural values, media, food availability, and nutrition knowledge—as well as internal factors—such as psychological development and characteristics, body image, values and personal experiences, self-esteem, food preferences, and health [3,6,7].

Despite the complex interplay involved in eating behavior, educational strategies that focus solely on the individual sphere of the child remain common in nutrition. However, behavioral changes are challenging, especially when they involve eating habits established since childhood [8]. Such changes require not only knowledge but also emotional support, consistency in the environment, and continuous reinforcement [9]. In this context, interventions involving the family unit appear to be favorable for maintaining healthy eating behaviors [10]. Studies indicate that positive parental practices—such as having family meals and involving children in food preparation—are associated with healthier diets, while negative approaches—such as pressuring children to eat or using food as a reward—may lead to unhealthy eating patterns [11,12,13].

Therefore, nutritional actions must be in the family context to promote lasting behavioral changes [13]. Furthermore, successful changes in eating behavior involve multicomponent approaches that combine nutrition education, family support, motivational strategies, and modifications in the food environment [14]. Active participation of parents and caregivers, as well as coherence between what is taught and what is practiced in the family environment, are determining factors for the success of these strategies [9,13,15].

Based on this premise, the evaluation of eating behaviors among children with overweight or obesity is important not only to describe dietary patterns but also to explore how behavioral changes are perceived, negotiated, and incorporated into everyday family contexts. While the relevance of family-based interventions is well documented, qualitative evidence on how children and families experience these interventions across different ecological contexts remains relatively limited. Anchored in Bronfenbrenner’s Ecological Systems Theory [10], this study seeks to understand the perceived transformations associated with a nutritional intervention within a health program, examining changes in eating behavior and in autonomy, critical awareness, body perception, and lifestyle, from the perspectives of the children and their families.

## 2. Material and Methods

**Figure 1:**
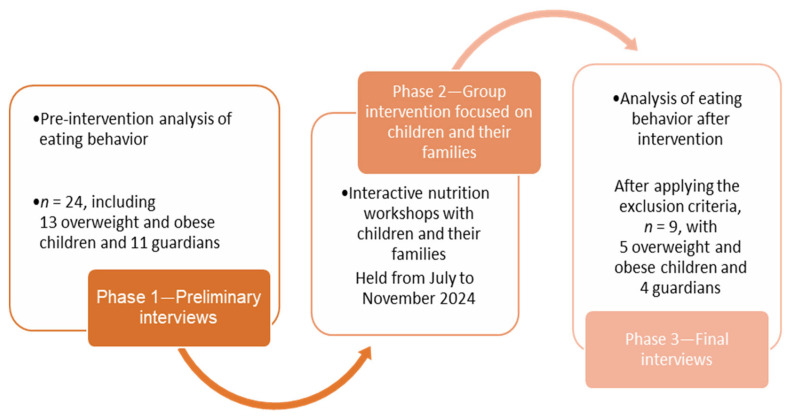
Stages of conducting the research. Source: The author (2023).

This study employed a qualitative approach and was conducted in three stages: 1. A preliminary semi-structured interview to assess eating behaviors before the intervention. 2. A group-based intervention involving children and their families. 3. A final semi-structured interview to evaluate eating behaviors after the intervention. The research stages are described in Figure 1.

This study was structured in accordance with the COREQ checklist [16], which aims to consolidate criteria for reporting qualitative research.

A pilot study was conducted to train the research team and test the applicability and clarity of the questionnaires, allowing for adjustments to procedures and ensuring higher data quality in the main study. Participants included children from the same target age group enrolled in the program, but with eutrophic BMI, and their families. During data collection, researchers recorded field notes, and after each session, they met to discuss challenges and propose adjustments. The revisions were incorporated into the final versions of the questionnaires and the operational protocol. Given the research team’s involvement in the design and implementation of the intervention, reflexive discussions were also undertaken throughout data collection and analysis to examine potential influences on interpretation.

### 2.1. Ethical Considerations

This research was approved by the Research Ethics Committee (CEP) of the Pontifical Catholic University of Paraná (PUCPR) under CAAE number 78754224.8.0000.0020 and opinion number 6.861.110. All investigations were conducted in accordance with the ethical principles of the Declaration of Helsinki of 1975, as revised in 2013.

All participating family members provided written informed consent for their own participation and for the participation of their children. Additionally, the children provided written assent prior to their inclusion in the study.

### 2.2. Context

The *ProSaúde Kids* program, linked to the Pontifical Catholic University of Paraná (PUCPR), was launched in August 2022 to promote healthy habits and improve the quality of life of children aged 3 to 12 years. The program is open to the public and has selection criteria, and in 2024 it had approximately 86 participants. Organized in age-based groups, sessions take place twice a week, combining physical education activities exploring various sports modalities with interventions from other fields—such as psychology and nutrition—scheduled on the same day as the sports classes. The model prioritizes a multidisciplinary and integrated approach focused on child development, promoting holistic care that considers physical, emotional, and social aspects. In addition, the program maintains affordable fees to encourage inclusivity.

### 2.3. Participants

The study population consisted of 51 children aged 3 to 12 years enrolled in the *ProSaúde Kids* program in 2023, in Curitiba, Brazil. Eligible participants were children aged 7 to 12 years who were classified as overweight or obese based on BMI-for-age, calculated using WHO AnthroPlus software (version 1.0.4; World Health Organization, Geneva, Switzerland), along with their families. The decision to include only children aged 7 years and older was due to the cognitive and verbal comprehension limitations of younger children (3–6 years), which could compromise their understanding and the validity of interview data.

Children were excluded if they: (i) missed any of the interviews (initial or final); (ii) withdrew from the *ProSaúde Kids* program before completion; or (iii) presented any neurodivergent condition that impaired comprehension at any stage of the study.

Parents or guardians were automatically included when the child met eligibility criteria and were excluded if they: (i) failed to attend both interviews; (ii) withdrew following their child’s dropout; or (iii) presented cognitive limitations or neurodivergence that impaired participation.

The initial sample included 13 children and their respective parents/guardians (*n* = 24). After applying exclusion criteria, the final sample comprised 5 children and their families (*n* = 9). One family (child + guardian) was excluded due to the absence of parents during the interview, and the remaining exclusions were due to early program withdrawal. Among the included children, four were female and one was male. Of the five guardians interviewed, two were fathers and two were mothers. No data on guardian age or socioeconomic status were collected, which limits contextual interpretation of the findings. None of the participants presented neurodivergent conditions that interfered with comprehension.

It is important to note that this study was embedded within the *ProSaúde Kids* program; therefore, participant recruitment and follow-up depended on children’s continuous enrollment in the program. Consequently, when families withdrew from the program, they were no longer available to continue in the research, directly contributing to the attrition rate and reduced final sample size.

This study did not seek to achieve theoretical saturation. Instead, all eligible children and families available and retained in the *ProSaúde Kids* program during the data collection period were invited to participate, and the final sample represents the total accessible population within this context. Although no a priori saturation criterion guided sample definition, data analysis indicated recurrence of themes across interviews, supporting analytical coherence within the study’s exploratory scope.

Despite the small sample, this size is consistent with the exploratory and in-depth nature of qualitative research, which prioritizes richness of data over numerical representativeness.

### 2.4. Research Phases

The planning and delivery of the interactive nutritional workshops, as well as the preparation of educational materials, were entirely carried out by the research team. The researchers also developed qualitative data collection instruments, including the interview guides. This full involvement allowed alignment between the study objectives, methodological strategies, and field implementation, consistent with qualitative research principles that recognize the researcher’s active role in knowledge production.

#### 2.4.1. Phases 1 and 3—Preliminary and Final Semi-Structured Interviews

Two sets of interviews were conducted with the children and their families: one before the nutritional intervention and another after its completion. The initial interview aimed to characterize pre-intervention eating behaviors, family eating routines, and consumption patterns. The final interview, conducted within two weeks after the last workshop, sought to assess participants’ perceptions and learnings from the intervention, exploring possible behavioral and attitudinal changes and how the workshop content had been integrated into daily life. The final interview revisited the same thematic axes as the initial one, adding questions to identify what had or had not been learned throughout the process.

Both interviews followed semi-structured guides developed based on Urie Bronfenbrenner’s Ecological Systems Theory of Human Development [5,6,12], considering its multiple systems. The interviews, lasting approximately 20 min each, included open-ended questions on general information, eating behaviors, and family food-related aspects.

Each interview was conducted using a printed guide and a notebook for audio recording. Before beginning, participants signed the informed consent form authorizing the use of audio and images for research purposes and the child assent form.

Interviews were conducted by a team of researchers who were not involved in the intervention phase and had no prior relationship with the *ProSaúde Kids* program to reduce bias and enhance neutrality. Two trained female health professionals from the field of dentistry conducted the interviews, ensuring standardized procedures and consistent data collection.

#### 2.4.2. Phase 2—Group Intervention for Children and Families

The intervention consisted of nine interactive nutritional workshops held between July and November 2024, on pre-scheduled days, focusing on health literacy [17] and using participatory methodologies [18,19] with family participation. The sessions took place in classrooms at PUCPR.

Each session was facilitated by three health professionals—two female and one male. They led interactive and educational activities promoting health and improving family eating behavior. Alignment among facilitators was maintained through asynchronous meetings to standardize practices and ensure methodological coherence. Health students from an interprofessional program also participated as observers, assisting with logistics and supporting children during activities.

The workshop themes were selected based on the *Brazilian Dietary Guidelines* [20] and the complementary literature, particularly those focusing on behavioral nutrition [5,21]. This approach allowed a broader understanding of eating beyond nutritional aspects, incorporating habits, culture, and social context—core principles of the *Brazilian Dietary Guidelines* [20]. The topics covered were: (1) “What is Healthy Eating”; (2) “Hunger for What?”; (3) “Food Selectivity”; (4) “Time to Go Grocery Shopping”; (5) “Autonomy in Choices”; (6) “Sharing Moments and Meals”; (7) “My Body Image”; (8) “Difficulties in My Child’s Feeding/Eating”; (9) “Applying What I’ve Learned”.

### 2.5. Data Analysis

Interview data were analyzed using the collective subject discourse (CSD) methodology proposed by Lefèvre and Lefèvre [22], which reconstructs collective thought from individual discourse synthesis. The analytical process followed the methodological stages defined by the authors: reading the transcripts, identifying key expressions, formulating central ideas (CIs), and constructing the final collective discourses (CSDs). For example, individual excerpts expressing children’s involvement in food choices (key expressions) were synthesized into central ideas related to autonomy, which were then integrated into collective subject discourses representing shared meanings across participants.

Interviews were transcribed using TurboScribe^®^ (TurboScribe Inc., San Francisco, CA, USA) and organized in Microsoft^®^ Word 2010 (Microsoft Corp., Redmond, WA, USA). Key expression identification and CI organization were performed using ATLAS.TI^®^ (version 23.4.0; ATLAS.ti Scientific Software Development GmbH, Berlin, Germany). To enhance analytical rigor and trustworthiness, two researchers independently conducted the initial stages of analysis without access to each other’s interpretations. Subsequent joint meetings were held to compare findings, resolve discrepancies through consensus, and refine the central ideas and final CSDs. This process supported reflexivity and consistency in qualitative interpretation. Analytical discussions focused on thematic recurrence, internal consistency, and alignment with the study objectives, rather than on achieving theoretical saturation, given the predefined and context-bound nature of the sample. In this context, formal member checking was not undertaken. Instead, credibility was enhanced through researcher triangulation, including independent analysis, consensus meetings, peer debriefing, and reflexive discussions.

The analytical lens was Bronfenbrenner’s Ecological Systems Theory of Human Development, which posits that sustainable changes in eating and lifestyle behaviors occur through the individual’s continuous interaction with multiple environmental systems [23]. The theory identifies interconnected systems—such as family, school, parents’ work, and the broader sociocultural context—that influence human development [10,23]. The definitions and descriptions of each system proposed by Bronfenbrenner’s theory are presented in Table 1.

## 3. Results

The central ideas (CIs) identified in the initial and final stages addressing the same topics were placed side by side in comparative tables to facilitate comparison of perceptions and reported behaviors across study stages. Table 2 presents the discourses of parents/guardians and children related to the microsystem, while Table 3 presents the collective subject discourses related to the mesosystem, focusing on interactions between family, school, and health professionals before and after the intervention.

At the microsystem level, caregivers and children described changes in family routines after the intervention. Children reported greater involvement in food-related decisions, particularly during grocery shopping. Increased openness to trying new foods, such as fruits and vegetables, was also described, and workshop content was shared with other family members.

Participants reported a gradual reorganization of meals, including perceived reductions in the consumption of ultra-processed foods and greater attention to dietary balance and satiety. Although not all changes were fully consolidated, families described progress in awareness and daily practices related to eating habits.

Although the nutritional intervention did not take place directly within the school environment, the mesosystem (particularly at the interface between school and family) remained relevant to children’s eating habits. The school maintained a structured eating routine, with fixed schedules and predefined menus, serving as the setting for many main meals, such as lunch and snacks. However, limitations imposed by this environment, such as the inability to change the school menu, remained unchanged in the post-intervention period. Despite this, changes were reported in the content of snacks brought from home, which were described as healthier, alongside greater family involvement in food-related practices.

Furthermore, the interaction among different environments—especially between school, caregivers, and other health professionals—continued to be described as influencing children’s eating behavior. Additionally, even without a formal school-based intervention, existing initiatives such as cooking classes and nutrition education activities were remembered positively by some families, serving as stimuli for children’s food autonomy. These school experiences, when combined with family involvement, were associated with children’s interest in healthier foods and cooking. Thus, even though the school’s structure itself was not altered by the intervention, its connection with the family context remained present in participants’ narratives about eating practices.

After the intervention, children and their families began to share the knowledge acquired about healthy eating with other family members and in external settings. Children reported teaching grandparents and peers about healthy eating habits, while parents mentioned applying the guidance they received within their social and professional circles.

Table 4 presents the collective subject discourses related to the exosystem, focusing on indirect influences on children’s eating behavior, such as media exposure and family work routines, before and after the intervention.

Before the intervention, participants reported frequent exposure to food-related content disseminated through media, especially the internet and television. Despite this exposure to recipes and dietary tips, participants described limited changes in the eating habits of children and their families. Information was described as being consumed without clear criteria for accuracy or practical application. After the intervention, participants reported changes in how media content was interpreted—particularly among children, who described questioning the reliability of information and expressing a more critical view.

Prior to the intervention, caregivers described demanding routines and work schedules as barriers to adopting healthier eating habits. Meal preparation was often reported as compromised, with frequent consumption of ready-made or ultra-processed foods chosen for convenience. After the intervention, although time constraints and routine challenges persisted, participants reported greater organization of family meals, including more structured planning and greater attention to food choices. Even though structural barriers remained, changes in daily meal organization and planning were described.

Table 5 presents the collective subject discourses related to the macrosystem, addressing broader sociocultural values, beliefs, and norms associated with food, body image, and childhood obesity before and after the intervention.

The results derived from the analyzed statements describe changes in children’s perceptions of food and body image after the educational intervention. Prior to the intervention, participants described concerns related to controlling children’s weight, often in the context of aesthetic standards and fears of obesity, alongside restriction of foods perceived as “fattening.” Food was also described as being used as an expression of affection or as a reward, with occasional allowances for less healthy foods in specific situations. In addition, children expressed dissatisfaction with certain body characteristics, which participants associated with external comments and beauty standards.

After the intervention, children described greater acceptance of body diversity and expressed more positive perceptions of their own bodies. Parents and caregivers reported reflecting on their feeding practices, seeking to balance the provision of healthy foods with moments of enjoyment—particularly on weekends—although some association between food and reward was still reported.

Table 6 presents the collective subject discourses related to the chronosystem, addressing changes and transitions in eating behavior over time and across different life stages before and after the intervention.

The collective discourses constructed from the chronosystem describe changes in eating practices over time. Prior to the intervention, participants reported a history of changes associated with life events, such as the birth of siblings or dieting phases among family members. Many parents mentioned that as their children grew older, adherence to healthy eating habits was described as declining, with frequent replacement of main meals by snacks or ready-made foods.

After the intervention, participants described greater awareness of how life stages influence eating habits, alongside reports of attempts to maintain a more balanced diet despite individual preferences within the family. The transition to adolescence was described as a challenge due to the pursuit of food autonomy, and participants reported reflections on the importance of healthy food choices and acceptance of bodily changes over time.

## 4. Discussion

Given the growing challenge that childhood overweight and obesity represent, understanding how nutritional interventions can promote changes in children’s eating behavior, especially when involving the family nucleus, becomes essential [24]. Within this context, the present exploratory study sought to examine how children and their families perceived changes associated with participation in a family-centered nutritional intervention. The study offers a contextual and descriptive perspective on eating practices and the environmental and relational factors that influence them. Through participants’ narratives, it was possible to observe perceived changes in eating behaviors and parental engagement in family routines. The following sections discuss the main findings, their implications for child health promotion, and potential directions for improving future educational strategies.

Regarding the family environment, perceived shifts in behaviors were described within the microsystem, as proposed by Bronfenbrenner’s ecological theory [10], including the reproduction of practices learned at home and greater parental awareness of their role in shaping children’s diets. The scientific literature supports the importance of the family as the central nucleus for the formation and dissemination of eating habits. Studies indicate that family members are the primary behavioral models for children and exert a strong influence on dietary habits from early childhood [25]. Moreover, healthy family practices can generate a multiplier effect, benefiting not only children but also the surrounding community [26]. In this sense, the findings align with the existing literature and point to the potential relevance of strategies that go beyond a child-centered focus, involve the family, and promote collaborative action among different professionals [13,27].

It was also observed that busy routines, long working hours, and multiple responsibilities make it difficult for parents to organize meals and offer healthier foods, often leading to quick and less nutritious choices. Eating behavior is influenced by various environmental factors, including family, socioeconomic, and cultural conditions, with the family being one of the main determinants in establishing healthy eating habits [27]. The literature reinforces that interventions involving families are more effective in promoting sustainable changes in children’s eating behavior, as they operate within the environments where these choices are constructed and reproduced [13,26,27].

Within the mesosystem, families reported perceived changes in school snacks, which became healthier, indicating possible greater family participation and responsibility in food-related decisions. Evidence shows that the school setting, including educational activities on healthy eating, can positively influence children’s nutritional status, provided that these initiatives are well implemented [28]. The inclusion of cooking workshops in schools—mentioned by participants—has been recognized as a low-cost, easy-to-implement, and highly applicable educational strategy in school contexts [29,30]. Moreover, such activities can be integrated into multiple curricular subjects, promoting learning in an engaging and practical way [29]. These effects add to positive parental practices, such as involving children in meal preparation and providing healthy options, which have a direct impact on their eating habits [12,13]. The present findings do not demonstrate such effects but suggest how families interpreted and integrated school-related food practices.

However, in Brazil, the presence of school canteens offering ultra-processed foods and the influence of food marketing around schools can undermine these efforts, highlighting the need for stronger regulation of the school food environment [31]. Although some legal initiatives exist, such as state and municipal regulations restricting the sale of ultra-processed foods in schools, e.g., Paraná State Law No. 14.855/2005 [32], in practice, these measures are often insufficient. Schools may comply with current regulations, but surrounding snack bars and candy stands still offer easy access to unhealthy products, since the legislation does not apply to them [33,34]. This gap compromises the progress achieved within schools and demonstrates the need for broader public policies that also encompass the school surroundings as part of children’s food environments.

Another finding from the collective subject discourses (CSDs) concerns the influence of media on children’s eating habits. Media content—such as advertisements and online videos—can shape food preferences, often promoting the consumption of ultra-processed and low-nutrient foods [35]. Our findings portray that media, particularly television and the internet, are frequent sources of information for both children and parents. Similarly, regarding media influence, children described a more critical understanding of food-related content after the intervention. These perceptions suggest increased awareness, reinforcing the potential value of incorporating media literacy into nutrition education strategies, as supported by previous studies [36].

Issues related to body image and emotional eating also emerged from the families’ narratives. The findings suggest that the intervention encouraged children to reflect more critically on their body image, showing signs of greater awareness and less influence from external beauty standards. This highlights the positive potential of integrated, developmentally sensitive approaches. Consistent with our findings, studies indicate that interventions addressing body image and eating behaviors can effectively promote healthy habits and build positive self-esteem among children and adolescents [25]. Similarly, educational actions that encourage critical thinking about media-driven beauty standards can reduce social comparison and body dissatisfaction, promoting greater autonomy and healthier eating behaviors [37].

The discourses also reflect how the time and significant life events affect the formation of children’s eating habits. Transitions such as the birth of siblings, changes in routine, or entry into adolescence were linked to altered eating behaviors, often associated with episodes of anxiety and emotional eating—an association well documented in the literature [38]. A systematic review and meta-analysis found that psychological stress in children and adolescents is associated with more unhealthy dietary patterns, including higher consumption of fatty and sugary foods and lower intake of fruits and vegetables [39]. Another meta-analysis identified a significant association between depressive symptoms and disordered eating behaviors in youth, showing that depression is positively related to emotional eating, suggesting that depressive symptoms may lead to harmful eating patterns triggered by negative emotions [40]. These findings emphasize the importance of interventions addressing not only nutritional aspects but also the emotional and psychosocial factors that influence eating behavior. Family-based strategies that promote emotional regulation may be more effective in fostering healthy eating habits and preventing long-term disordered eating patterns.

Some limitations must be acknowledged in this article. As a short-term study, it was not possible to evaluate whether the reported changes were maintained over time. The analysis also relied on self-reported data, which may be subject to bias, particularly social desirability bias [41], where participants may have sought to please researchers by giving more positive responses about the program. Although this tendency was mitigated through the use of external interviewers who were not involved in the intervention, it cannot be completely ruled out. In addition, the absence of objective nutritional indicators and the lack of socioeconomic characterization of participating families restrict the scope of interpretation and limit a more contextualized understanding of the findings.

The small sample size also limits the transferability of the findings, as results may not fully represent the diversity of experiences among children with overweight and obesity. This limitation is partly related to the context in which the study was conducted, as the research was embedded within an ongoing health promotion program. Participation in the study depended on children’s continued enrollment in the *ProSaúde Kids* program; therefore, withdrawal from the program necessarily resulted in discontinuation from the research, contributing to the high attrition rate and reduced final sample size. In addition, limited engagement from some children during interviews represented another challenge, as responses were occasionally brief or underdeveloped, possibly due to shyness, comprehension difficulties, or discomfort with the interview setting. This limitation may have affected the depth and diversity of children’s perspectives captured in the data.

Taken together, these limitations indicate that the findings should be interpreted as context-specific and exploratory rather than generalizable. Therefore, future interventions should employ mixed-method designs with more robust quantitative and qualitative components and include medium- and long-term follow-up strategies. Longitudinal studies, for instance, could assess the maintenance of acquired habits and provide insight into the long-term effects of educational interventions. Moreover, incorporating systematic evaluation mechanisms for the school food environment and the role of media in shaping eating behavior could broaden the scope and impact of such programs.

Despite these limitations, the results of this exploratory study suggest the relevance of educational spaces that integrate family and community, highlighting the potential role of the family environment in building healthy habits. By encouraging reflection and mindful eating practices, the intervention appeared to offer opportunities to promote positive changes in children’s eating behavior and their relationship with body image. Furthermore, the use of participatory methodologies can be seen as a valuable approach to promote active listening and the involvement of children and families in the educational process, which can increase engagement, autonomy, and the meaning of the proposed changes. In this sense, the study points to the potential value of strategies that encourage family participation and shared responsibility in food-related decisions, as well as the relevance of integrated actions between health, education, and other sectors that shape children’s social environments. Overall, the contribution of this study is primarily contextual and descriptive, offering exploratory insights that may inform future research and practice, without implying causal effects or generalizability.

## 5. Conclusions

Our findings suggest that this exploratory intervention was associated with perceived changes in the eating behavior of children and their families, including increased reflection on food choices and everyday practices. Children reported greater critical awareness of body image and food-related media content, indicating possible reductions in the influence of external stereotypes and media standards, and suggesting that the educational experience may have extended beyond nutritional aspects alone.

While these findings should be interpreted in light of the study’s qualitative design, small sample size, and reliance on self-reported data, the results point to the potential relevance of strategies that acknowledge the complexity of social and cultural contexts involved in shaping eating habits. In this sense, the study contributes exploratory insights that may inform the development of family-centered and context-sensitive approaches to health promotion in childhood, without implying generalizable or causal effects.

## Figures and Tables

**Table 1:** Thematic nuclei defined by Bronfenbrenner’s theoretical model.

System	Definition
Microsystem	The child’s most proximal environment, involving direct and frequent relationships (family, school, health professionals, neighborhood). This is where direct interactions that affect eating behavior occur: family habits, eating practices within the home, parental influence, dialogue with nutritionists, and school experiences related to food.
Mesosystem	Refers to the articulation between proximal environments, such as the relationship between the family and the school, or between caregivers and health professionals.
Exosystem	Environments in which the child does not participate directly, but that affect their development. Indirect factors, such as parents’ work schedules which limit the preparation of healthy food, food access policies, and media influences (commercials, social media).
Macrosystem	The broader sociocultural context—values, beliefs, ideologies, cultural norms. It includes cultural beliefs about food (food as affection, rewards), body stereotypes, beauty standards, and social norms that shape perceptions of childhood obesity and healthy eating.
Chronosystem	The time dimension—changes and transitions throughout life. It encompasses the transformations in eating behavior over time. It also considers significant life events that impact eating habits.

Source: The author (2025).

**Table 2:** Collective subject discourses, from caregivers and children, before and after the intervention, regarding the microsystem theme.

THEME—MICROSYSTEM	
Brief explanation of the theme: The child’s most proximal environment, involving direct and frequent relationships (family, school, health professionals, neighborhood). This is where direct interactions that affect eating behavior occur: family habits, eating practices within the home, parental influence, dialogue with nutritionists, and school experiences related to food.	
**Initial discourse**	**Final discourse**
**CI 1—Children’s participation in food decisions**	
**Caregivers**	
**DSC:** We decide. Sometimes on the weekend, they want beef parmigiana. But that’s a rarer thing to make, usually it’s just regular beef, with onions, a little fat, nothing too greasy. Sometimes whatever they feel like, but it’s almost always the same things.	**DSC:** She/He helps in the kitchen, already packs her/his own lunchbox. I put what is on the table, and everyone chooses what they will take, what quantity. Over the last year, she/he has been changing. She/He is more interested.
**Children**	
**DSC:** My mother, my grandmother, or my father choose what we are going to eat. At home, Mom makes normal things, it’s nothing too different. If you don’t want to eat what’s there, you don’t come. I help sometimes, when she (Mom) asks to cut salad, I help her put soap in the washing machine, hang the clothes, but not in the kitchen.	**DSC:** We help with the shopping, we do several things. Sometimes I make it, sometimes I make cookies by myself. Now, you see what is on the table: rice, beans, polenta, meat, and salad, and you take what you want. When she puts what we have for lunch, like rice, beans, and meat, I choose something else, like the salad with rice and meat. I like to cook, but I like to cook sweets, not savory food.
**CI 2—Introduction and acceptance of healthy foods**	
**Caregivers**	
**DSC:** For her/him, we always put a little bit on the plate and say that she/he has to eat and try it. Then she/he says, I can’t, I don’t like it. We try salad, but there is resistance, melon, she doesn’t like to eat much of it. Sometimes, if she sees a little chopped onion, she/he doesn’t want to eat it. But it tastes the same, she just can’t feel the texture. I don’t use a technique, but I put it on the plate and say she has to try it.	**DSC:** This rule of trying it 10 times, I think it’s classic, wonderful, and it absolutely works great at home, yes. And it’s not just about food, it’s about everything in life. So that you can have an opinion about it later, right!? He started accepting more easily, she/he tried it.
**Children**	
**DSC:** I don’t like to try food, I think it’s boring. I kind of like to try new foods [shakes head no].	**DSC:** I learned to try things, I like it, but Mom always makes new things, so I’m not always trying, we have an agreement that we have to try it 10 times to say we don’t like it or that we love it, so I like to try new foods. I think after the classes I stopped judging so much by the appearance, smell, and color of the food and more by its taste. In class, there were several little bowls with lettuce, grated carrot, there was broccoli, we even had to pick it up, pass it on our tongue to feel what it’s like, smell it, make a little sound like that, like see if it makes a noise, and we made an agreement that we have to try it 10 times to say we don’t like it or that we love it, so I like to try new foods.
**CI 3—Consumption of ultra-processed foods and changes in family shopping**	
**Caregivers**	
**DSC:** She/He usually prepares bread, with cheese and ham. The part about sweets, sugar, those things, she/he always had access to the grandparents, but also started eating sweets because I used to sell them. We try to control the diet more, but it’s not always so easy. The instant ramen (“miojo”), which she/he also likes very much, I think that’s the worst, and sometimes, she/he makes it secretly, which I’ve noticed, pancake with sweet filling.	**DSC:** I buy more salad and less junk food, more yogurt, more fruit, he likes grapes a lot, so I eat a lot of fruit for her/him too. This improved. Cookies, sweets, candies, those things that we sometimes always brought home in the shopping for them, I cut them out completely. I don’t buy them anymore. She/He understood that she/he won’t eat a little chocolate every day, and oh, I think we learned a little more about… Not that we didn’t know, but that we didn’t put it into practice. We sometimes ate a lot of ultra-processed foods, but now we don’t eat them much anymore. And now, they learned about that magnifying glass thing. Now, they check everything.
**Children**	
**DSC:** When I go to the supermarket, I like to buy sweets, candies, cornflakes (“sucrilhos”), sweet biscuits (“sequilhos”)… I like to choose mini-hot dogs (“doguinho”) at the supermarket. In the morning, I have chocolate milk powder drink (“nescau”) and cookies. I like to eat potato chips. While I eat, I drink tea, soda…	**DSC:** At the supermarket, sometimes I choose bread, a cake, or a sweet, but there is a rule. When you see a magnifying glass, you know you shouldn’t buy it. Because it has a magnifying glass, it means there’s a warning. We stopped drinking beverages with food a little, and we also stopped buying soda and tea a little.
**CI 4—Influence of grandparents and extended family on eating practices**	
**Caregivers**	
**DSC:** The part about sweets, sugar, those things, she/he always had access through the grandparents, and it ended up with the child, you know how it is, right, they like those things. When they go to their grandparents’ house on the weekend, they always eat them. The grandparents are totally contrary to us.	**DSC:** What they acquired in terms of sweets is through the grandparents, but they also already understood this process. They say to my mother: Grandma, the Magnifying Glass, Grandma. And then my mother says: Oh, so we must do it like this now. Oh, we have to do it like that. So, they become more vigilant.
**Children**	
**DSC:** Sometimes, my grandmother makes the food. When I want something, I go to the supermarket with her, because then I paid with my money, which I got as an allowance from my grandmother.	**DSC:** Grandma and Grandpa sometimes go to the bakery, they buy some savory snacks too, cheese bread, but we explain everything we learned to Grandpa and Grandma.
**CI 5—Organization of the family eating routine**	
**Caregivers**	
**DSC:** There is no planning, we decide what we are going to do at the moment. Sometimes she/he wants a sandwich, sometimes we make a snack, something like that. Only my husband’s food is what’s on the doctor’s menu, but me and her/him, we decide, sometimes we feel like it, but it’s always almost the same things.	**DSC:** We have already organized ourselves. Of course, there are days when it works perfectly. There are days when it doesn’t work, right? The program contributed with small things. But in the end, they make a big difference. The meal is just us, at the table just us. We also agreed that there is no plate reproduction at night (no second helpings).
**Children**	
**No perceptions**	**No perceptions**
**CI 6—Practices during meals: screens, pace, and body awareness**	
**Caregivers**	
**DSC:** Sometimes it’s a fight, one wants to eat on the sofa, another in the bedroom, and I always insist on eating at the table, right? But, sometimes, they go out to the bedroom, but then, sometimes, I shout a bit, they come back to the table. Usually, the TV is on, but it doesn’t end up being watched, it’s just for having some sound on. The cell phone is rare, so much so that sometimes I question her/him to put it away, but my husband checks it a little, sometimes I check it a little. It’s not like, that addiction, where they are on the phone all the time during the meal. Sometimes even next to them, sometimes they check it quickly, but they are not glued to it all the time during the meal, no. I always try to stop her/him, sometimes we end up doing it wrong, but I try to make sure she/he doesn’t do the same.	**DSC:** Sometimes we eat watching television, but we also talk while we eat. I think eating has become more conscious. I think I changed that word: “I’m full,” no, “I’m satisfied.” So, maybe you are already satisfied? So, it’s truly a movement of awareness, of becoming conscious, and that makes a difference.
**Children**	
**DSC:** Most of the time, we eat watching television, but we also talk. At breakfast, since I’m alone, I stay on my cell phone watching my series.	**DSC:** Sometimes we eat watching TV or when we eat pizza, sometimes they talk and I listen. I think it also changed a little regarding chewing, before I chewed very little, now I chew a little more.

Source: The author (2025).

**Table 3:** Collective subject discourses of the central ideas regarding the mesosystem theme.

THEME—MESOSYSTEM	
Brief explanation of the theme: Refers to the articulation between proximal environments, such as the relationship between the family and the school, or between caregivers and health professionals.	
**Initial discourse**	**Final discourse**
**CI 7—Interaction between school and family in child feeding**	
**Caregivers**	
**DSC:** She/He spends most of her/his time at school, right? But in the evening we are closer. She/He has lunch at school, however, she/he takes the lunchbox we make at home. She/He takes a snack in the morning, has lunch at school, where she/he eats what she/he wants, and takes another snack in the afternoon. The school has helped a lot, but when there’s a school party, she/he already wants to eat a certain thing.	**DSC:** She/He spends most of the day at school. She/He has lunch and a snack at school. The food at the school is more balanced, so much so that she/he even has cooking classes at school. They take their own lunchbox, which is also healthy. I found it very interesting that they are having nutritional education classes that they didn’t have before, so, I found it quite interesting.
**Children**	
**DSC:** I eat at school, I have lunch at school, with my friends. Usually it’s risotto and ponkan, or banana. There at our school, sometimes it’s different, like, pizza… But the menu is on the calendar.	**DSC:** I eat at school. At school, there is a snack that the school provides or you bring your own, but usually, we bring something. There is also fruit at school. The snack, I think, is around two forty, since recess is at three thirty, right?
**CI 8—Influence of health professionals**	
**Caregivers**	
**DSC:** My daughter/son received nutritional guidance through the insurance plan, I don’t know where, but she/he didn’t like it very much. She/He found the activities they did with her/him not interesting.	**DSC:** Ah, after the nutrition program here, I understood, I buy more salad and less junk food. When she/he decides to make a change, we remember the nutritionist here. I think addressing this issue with the children in this way was good. So, something more dynamic, like this year, was more interesting.
**Children**	
**DSC:** I’ve already been to a nutritionist through the plan, I thought it was okay, but I don’t know why. The doctor said I had to eat differently. I can’t eat a lot of instant ramen (“miojo”), fermented milk drink (“Yakult”), and other things, but sometimes I eat them.	**DSC:** After the nutrition class here, we realize that we can’t eat rice, beans, pasta, and potatoes on the same day. It was fun. Here I learned to try things and I ate broccoli.
**CI 9—Multiplication of knowledge: the child and the family as agents of influence in other environments**	
**Caregivers**	
**No information**	**DSC:** I think besides at home, I ended up using some things in my office. I always tell my sister; she has three girls and is going to start participating in the project next year. I don’t pass it on to other people, but I comment on it, yes.
**Children**	
**No information**	**DSC:** I teach some things. We explain everything to Grandpa and Grandma and we pass some things on to other people. At home, we eat a lot of potato, sweet potato, mashed potatoes along with rice, pasta, then I just explained that it’s not very good, because both are carbohydrates.

Source: The author (2025).

**Table 4:** Collective subject discourses of the central ideas regarding the exosystem theme.

THEME—EXOSYSTEM	
Brief explanation of the theme: Environments in which the child does not participate directly, but that affect their development; indirect factors, such as parents’ work schedules which limit the preparation of healthy food, food access policies, and media influences (commercials, social media).	
**Initial Discourse**	**Final Discourse**
**CI 10—Media and digital content as influencers on diet**	
**Caregivers**	
**DSC:** We always end up reading something on the internet, or seeing it on TV, right? I get tips and recipes on the internet, but I fail a lot with this, I don’t always end up following it. I also look for ideas and courses on the internet …	**DSC:** Sometimes I see it on the internet, sometimes on television. We follow some famous people on the internet. And always thinking about making healthy things, about exchanging and adapting. We also have little books; they must have half a dozen or ten children’s recipe books, with healthy recipes.
**Children**	
**DSC:** I’ve seen recipes on the internet. [Nods head]. I’ve also seen it on the news, on the internet, or with my grandma, but it hasn’t changed my diet.	**DSC:** I see advertisements and recipes on the internet, saying this is good, this is bad, or a recipe. It’s something I see a lot about food on the internet, but I haven’t changed much. In the classes here, I learned that many times most of the things we see on the internet are not true.
**CI 11—Family routine and work schedules as barriers or facilitators**	
**caregivers**	
**DSC:** The rush of the day and work hours make us choose the most practical option. Many times there’s no time to prepare a healthier meal, so we end up resorting to what is quicker. Due to the rush, there was a lot of ready-made food; we even had it ready to make things easier. Ready-made meals, for example, yakisoba, we would order ready. It’s difficult, you see, to quit iFood. I didn’t have time. Then, I reduced my workload at work, to take care of this part, right? Because it was very messy.	**DSC:** Everything is very rushed, there is a lot of work and everything, but it is healthier, now I try to organize better. But, day-to-day, it really is a challenge. There are more things that we organized better. Of course, there are days when it works perfectly. There are days when it doesn’t work, right?
**Children**	
**No information**	**No information**

Source: The author (2025).

**Table 5:** Collective subject discourses of the central ideas regarding the macrosystem theme.

THEME—MACROSYSTEM	
Brief explanation of the theme: The broader sociocultural context—values, beliefs, ideologies, cultural norms. It includes cultural beliefs about food (food as affection, rewards), body stereotypes, beauty standards, and social norms that shape perceptions of childhood obesity and healthy eating.	
**Initial Discourse**	**Final Discourse**
**CI 12—Concern about weight and childhood obesity as a reflection of aesthetic and health values**	
**Caregivers**	
**DSC:** I am very afraid that my daughter/son will become obese. We worry because we know that excess weight can cause health problems and also difficulty in mobility, and she/he has always been overweight. I myself have this tendency, so I try to control it through diet. I avoid certain foods precisely for this reason, because I know she/he can gain weight easily, we take care, then we end up relaxing and it comes back.	**DSC:** He even says he is losing a little weight, he is quite chubby. (Less explored by parents)
**Children**	
**DSC:** My body was better before because my belly was small. And my mother and I started going to the gym. My mother was also a little overweight. When I look back, I feel my body was better than before because my belly was smaller, sometimes I get upset about this.	**DSC:** I like my body. I wanted to be taller. In the nutrition class, we did several activities about the body and our appearance, and I think that also helps with self-esteem because no one is the same as anyone else, no one needs to base themselves on or compare themselves with others.
**CI 13—Food as an expression of affection, reward, or permissiveness**	
**Caregivers**	
**DSC:** Even with concern about weight, sometimes we end up giving in. On the weekend, there is always that little treat. We always avoid these things, like chips, those things, I don’t let them eat, but once in a while, also… She/He likes sweets during the week, but more on the weekend we allow some kind of different food. We try to control the diet more, but it’s not always so easy.	**DSC:** On the weekend, I have to have the little treat, but now I try to balance it more. The weekend is always together, sometimes we order pizza and Saturday is the day to eat savory pastries (“pastel”). Usually, after meals, we drink water. On the weekend, we open a soda. The consumption used to be higher, you know?
**Children**	
**No information**	**No information**

Source: The author (2025).

**Table 6:** Collective subject discourses of the central ideas regarding the chronosystem theme.

THEME—CHRONOSYSTEM	
Brief explanation of the theme: The time dimension—changes and transitions throughout life. It encompasses transformations in eating behavior over time. It also considers significant life events that impact eating habits.	
**Initial Discourse**	**Final Discourse**
**CI 14—Dietary changes over time**	
**Caregivers**	
**DSC:** As time went by, our diet changed quite a bit. When the children were small, we took better care, but nowadays it’s harder to maintain a healthy routine. After the birth of the sibling, who is now six years old, she became even more anxious, as if she felt more insecure inside the house; she has already improved a lot, but today the connection she has with food is with this feeling. We also used to cook at home before, but today we sometimes end up eating snacks or ordering food. We also had phases where we tried to change, like when someone in the family went on a diet, but then we returned to the previous habits.	**DSC:** We try to maintain a routine with more healthy foods. I won’t say that I managed to change completely, because at home there is a child, there is a pre-teenager, and there is an adolescent and an adult child. So, they are in different phases and each one wants to eat something different, but it has helped.
**Children**	
**No information**	**No information**

Source: The author (2025).

## Data Availability

The data presented in this study are available on request from the corresponding author due to ethical and privacy restrictions.
